# Curative Effects of Copper Iodide Embedded on Gallic Acid Incorporated in a Poly(vinyl alcohol) (PVA) Liquid Bandage

**DOI:** 10.3390/gels9010053

**Published:** 2023-01-08

**Authors:** Putita Phetcharat, Pakakrong Sangsanoh, Chasuda Choipang, Sonthaya Chaiarwut, Orawan Suwantong, Piyachat Chuysinuan, Pitt Supaphol

**Affiliations:** 1 The Petroleum and Petrochemical College, Chulalongkorn University, Bangkok 10330, Thailand; 2 Research Unit on Herbal Extracts-Infused Advanced Wound Dressing, Chulalongkorn University, Bangkok 10330, Thailand; 3 School of Science, Mae Fah Luang University, Chiang Rai 57100, Thailand; 4 Center of Chemical Innovation for Sustainability (CIS), Mae Fah Luang University, Chiang Rai 57100, Thailand; 5 Laboratory of Organic Synthesis, Chulabhorn Research Institute, Bangkok 10210, Thailand

**Keywords:** poly(vinyl alcohol) (PVA), cuprous iodide (CuI), green synthesis, liquid bandage

## Abstract

In daily life, people are often receiving minor cuts due to carelessness, leaving wounds on the skin. If wound healing is interrupted and the healing process does not finish, pathogens can easily enter wounds and cause infection. Liquid bandages are a fast and convenient way to help stop the bleeding of superficial wounds. Moreover, antibacterial agents in liquid bandages can promote wound restoration and fight bacteria. Herein, a poly(vinyl alcohol) (PVA) liquid bandage incorporating copper iodide nanoparticles (CuI NPs) was developed. CuI NPs were synthesized through green synthesis using gallic acid (GA) as a reducing and capping agent. The sizes of the CuI NPs, which were dependent on the concentration of GA, were 41.45, 43.51 and 49.71 nm, with the concentrations of gallic acid being 0, 2.5 mM and 5.0 mM, respectively. CuI NPs were analyzed using FTIR, XRD and SEM and tested for peroxidase-like properties and antibacterial activity. Then, PVA liquid bandages were formulated with different concentrations of stock CuI suspension. The results revealed that PVA liquid bandages incorporating 0.190% CuI synthesized with 5.0 mM of GA can kill bacteria within 24 h and have no harmful effects on human fibroblast cells.

## 1. Introduction

Damage to the skin makes the human body vulnerable to pathogenic invasion. Pathogens colonize their hosts and penetrate deeper tissues. As a result, wound healing can be disrupted and delayed. Therefore, methodical wound dressing to cover wound sites is a traditional approach to prevent bacterial wound infection. Currently, various cyto-compatible polymers, especially synthetic polymers, are widely applied for wound bandage formulation and several biomedical applications. Examples of synthetic polymers used for wound dressing are poly(vinyl alcohol) (PVA), poly(lactic-co-glycolic acid) (PLGA), polylactide (PLA), polyurethanes (PUs), poly(ethylene oxide) (PEO)/poly(ethylene glycol) (PEG), poly(hydroxyethyl methacrylate) (PHEMA), and poly(vinyl pyrrolidone) (PVP), due to their excellent biocompatibility, which makes them non-toxic to human cells. Additionally, most of these have been synthesized in 3D network hydrogels, which are a main focus of biomedical research [1,2].

Ideal wound dressings must have an appropriate water vapor transmission rate (WVTR), capability to provide moisture, suitable temperature and humidity to enhance wound healing, gaseous permeation, excellent antimicrobial properties, strong mechanical performance and the capacity to deliver active agents [3]. Poly(vinyl alcohol), a conventional synthetic polymer, has long been used for biomedical applications due to its non-toxicity to mammalian cells, biodegradability and biocompatibility. PVA has a large amount of hydroxyl groups in its structure, which provide a hydrophilic structure and sufficient swelling capacity, allowing it to absorb exudation to stop bleeding. Poly(vinyl alcohol) wound dressings, which are also popularly used in commercial products, are liquid gel bandages with quick film formation to prevent infection. In addition, PVA thin film is breathable to allow the exchange of gas and moisture in the wound, providing an optimal environment for wound healing [4,5].

Despite the fact that PVA is widely used for liquid bandages, it does not have inherent antibacterial activity because of its chemical structure. Therefore, active bacteria-killing agents play a significant role in PVA wound dressings in the production of alternative antibacterial bandages. Presently, there is various research reporting on polymeric materials containing metal nanoparticles to promote the wound healing process. It is widely known that metal NPs have significant potential as antibiotics [6,7,8]. Some reports have shown the strong ability of cyto-compatible polymers to kill bacteria when fabricated with silver nanoparticles or gold nanoparticles, due to their small surface-area-to-volume ratio [2,9,10]. Copper nanoparticles and copper-based compounds have been employed as practical materials in human tissues for centuries and have been used in biomedical and pharmaceutical applications since early 2005 [11]. They have been proven to be toxic to several bacterial strains through various mechanisms, including ROS production, metal ions released from the metal surface and denaturation of the biomolecules in bacteria [12,13,14]. Copper (I) iodide or cuprous iodide has been used as an active agent in wound dressings for its significant antimicrobial activity compared to other copper (II) or cupric compounds [15]. The reaction kinetics of copper compounds kill bacteria in a manner similar to that of natural enzymes in the human body, making them more attractive for the development of nano-enzyme antibacterial systems [16]. However, suitable content and crystallite size of metal nanoparticles should be considered for these materials to be safely applied to wound sites. Otherwise, they may be harmful to cell tissue. 

Various research has reported on diverse methods of controlling the crystallite size of synthesized copper iodide, such as the chemical route, co-precipitation and the microwave-hydrothermal assisted method. However, these syntheses require hazardous raw materials, complicated synthetic steps and high temperature, and are time consuming, resulting in high cost and biological harm. Consequently, less toxic processes are essential for copper iodide synthesis [17]. Green synthesis or biosynthesis is an eco-friendly method proposed to eliminate toxic and polluting chemical agents, consume less energy and allow the use of green solvents (water, ethanol, ethyl acetate, etc.) [17]. In addition, in green synthesis of copper iodide, the size of metal nanoparticles can be controlled by varying the concentrations of biochemical substances [18]. The usage of green chemical substances to synthesize copper iodide nanoparticles has become an extensive field, since they provide much greener and ecologically friendly routes of synthesis. Gallic acid (GA) is a well-known natural antioxidant derived from several plants, such as berries, fruit and tea. Gallic acid is a type of phenolic compound that reduces inflammation and oxidative stress damage. There are several reports on the use of gallic acid as a bio-stabilizer and bio-reducer in the synthesis of functional nano-enzyme materials [19]. By varying the concentration of gallic acid, one can characteristically produce a wide range of nanoparticles and control their size. 

In this work, we study the therapeutic effects of antibacterial liquid bandages produced with gallic acid–copper iodide nanoparticles (GA-CuI NPs) incorporated in poly(vinyl alcohol) through ultrasonic homogenization. The appropriate content of GA-CuI NPs to load into PVA liquid bandages was investigated. The physical and chemical properties of PVA liquid bandages with different concentrations of CuI suspension were characterized by various methodologies, including FTIR, XRD, FESEM, drying time and water vapor transmission rate. In addition, the biological properties of synthesized liquid bandages were tested in vitro for cytotoxicity to human dermal fibroblast cells and antibacterial activity against *E. coli* (Gram-negative) and MRSA (Gram-positive) to confirm their practicality for use in biomedical applications.

## 2. Results and Discussion

### 2.1. Encapsulation and Stabilization of Gallic Acid–Cuprous Iodide Nanoparticles

The synthesis of cuprous iodide (CuI) nanoparticles that is stabilized and reduced by gallic acid is a simple and convenient approach of green chemistry. Green synthesis of metallic compounds is rapid and highly effective when using phytochemicals, which are bioactive molecules from plants (such as gallic acid (GA)), as reducing and stabilizing agents, because they have abundant hydroxyl groups, phenolic groups and carboxylic groups, which are beneficial for capping metallic components. Several studies have shown that gallic acid concentration affects the size of metallic nanoparticles due to its bifunctional nature, i.e., its enol form and keto form [20,21,22]. Gallic acid is subjected to a two-electron oxidation process with two hydroxyl groups, which converts the enol form to the keto form. This formation of keto(quinone) enhances the interaction with the cuprous iodide nanoparticles and stabilizes the negative carboxyl group. 

#### 2.1.1. X-ray Diffraction (XRD) Analysis

**Figure 1:**
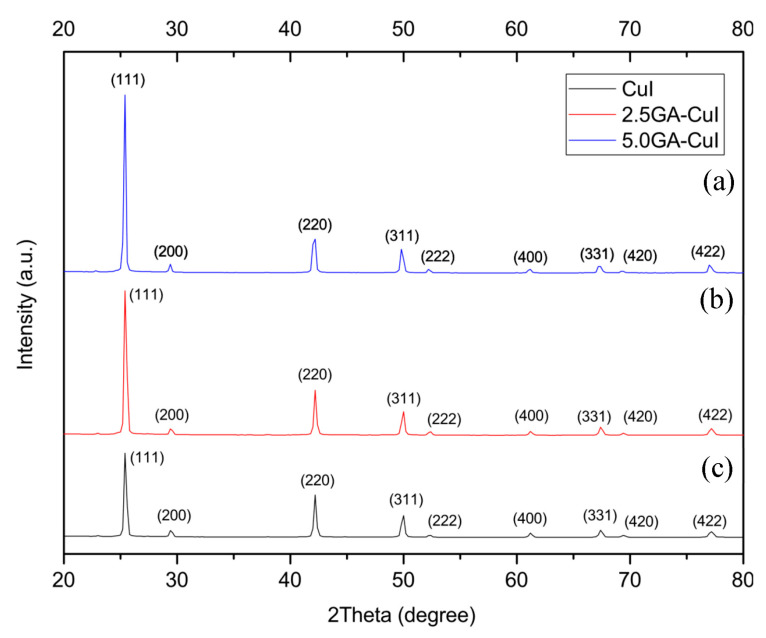
XRD patterns of synthesized nanoparticles (**a**) 5.0GA-CuI NPs (**b**) 2.5GA-CuI NPs and (**c**) CuI NPs.

The crystalline structure and phase transition of synthesized CuI NPs and GA-CuI NPs were investigated using X-ray diffraction (XRD) analysis. Figure 1a shows the XRD pattern of CuI NPs synthesized without gallic acid. The crystal lattice of CuI is reported to crystallize in a face-centered cubic (FCC) lattice; the γ phase is shown because it is the most stable structure at room temperature. The XRD pattern of CuI powder was found to relate to the database card numbers 01-089-7072 and 01-077-9397. Detailed peaks were noticed at the 2ϴ values 25.43°, 29.42°, 49.80°, 52.30°, 61.20°, 67.34° and 77.12°, correlating to the (111), (200), (220), (311), (222), (400), (331), (420) and (422) planes, respectively. These XRD peaks correspond to CuI synthesized using black soybean [23]. The crystallite size was calculated using Scherrer’s equation; the average crystallite size was found to be 41.45 nm, which is in the nano-size range.

$$t=\frac{K\lambda }{\beta \cos\theta }$$

t = crystallite size;
K = shape factor (0.9);
λ = wavelength of x-ray (0.154 nm);
β = full width at half maximum (FWHM);
ϴ = diffraction angle.

The crystal structure of cuprous iodide (CuI) synthesized using different concentrations of gallic acid as a stabilizing and reducing agent was also studied by X-ray diffraction (XRD). The XRD patterns of CuI stabilized with 2.5 and 5.0 mM of gallic acid are illustrated in Figure 1b,c. The well-defined peaks of both 2.5 GA-CuI and 5.0 GA-CuI correspond to JCPDS card number 82-2111, space group: F-43 m [24] and database card numbers 01-082-2111, 01-083-1106 and 01-075-0832, presenting the FCC unit cell. In the case of 2.5 GA-CuI NPs, peaks were observed at the 2ϴ values 25.46°, 29.46°, 42.20°, 49.8°, 61.20°, 67.38° and 77.08°, corresponding with the (111), (200), (220), (311), (222), (400), (331), (420) and (422) planes, respectively. The average size of 2.5 GA-CuI NPs is 43.51 nm. XRD peaks of 5.0 GA-CuI NPs were detected at 2ϴ values 25.42°, 29.44°, 42.25°, 49.83°, 61.10°, 67.22° and 77.03°. These peaks were matched with the (111), (200), (220), (311), (222), (400), (331), (420) and (422) planes, similar to the previous CuI NPs. The mean crystallite size of 5.0 GA-CuI is 49.71 nm. In addition, some lattice planes of CuI synthesized with different concentration of GA, especially (220), (311), (222) and (422) planes, demonstrate an insignificant shift. The 5.0 GA-CuI represented by the blue line shows a minor shift to a lower angle compared to CuI and 2.5 GA-CuI. This result is attributable to the larger crystallite size of 5.0 GA-CuI, which broadens the lattice planes and causes the lower angle shift. This result matches earlier research on the different sizes of synthesized gold nanoparticles [25]. 

According to the XRD patterns and different crystallite sizes of synthesized CuI NPs due to varying gallic acid concentration, it is clear that the gallic acid concentration is essential for controlling the size of these CuI NPs under normal pH conditions (pH~7). The size of CuI NPs increases with greater concentrations of gallic acid [20,26]. This result may be attributed to the higher negative charge of gallic acid, which remains on the copper surface and creates a repulsive force in the copper complex. The presence of this negative charge is due to the carboxyl group of gallic acid attached to the copper nanoparticle surface, and it provides stability through the electrostatic repulsion of CuI NPs in the solution.

#### 2.1.2. X-ray Photoelectron Spectroscopy (XPS) Study

**Figure 2:**
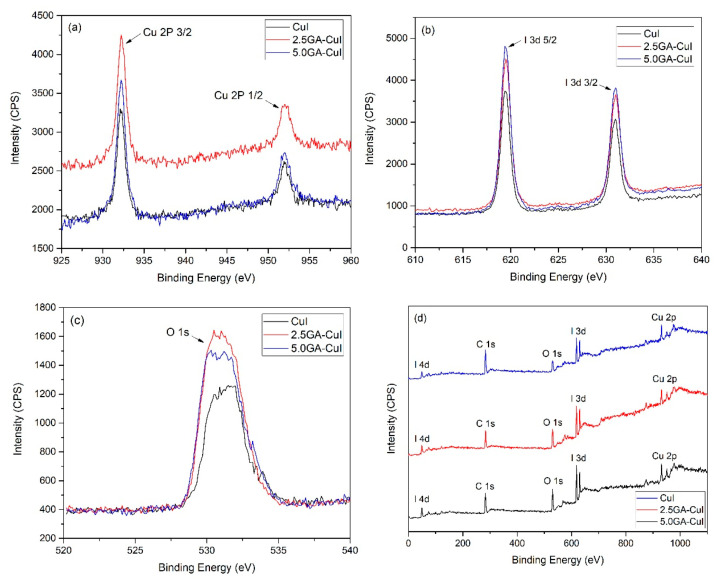
XPS spectra of (**a**) Cu 2p, (**b**) I 3d, (**c**) O 2 s and (**d**) survey spectra of CuI NPs.

The chemical state of synthesized CuI NPs was characterized by XPS analysis, presented in XPS spectra (Figure 2). The XPS spectra were scanned using adventitious carbon (C 1 s–284.6 eV) as the reference and used to investigate the exact oxidation state of the elements composing CuI NPs. Figure 2a shows the XPS peaks of the Cu 2p orbital. It can be noted that the binding energies around 932 eV and 952.4 eV belong to Cu 2p_3/2_ and Cu 2p_1/2_, respectively. Figure 2b illustrates the binding energies at 619.4 eV and 631 eV, which correspond to the I 3d_5/2_ and I 3d_3/2_ orbitals, clearly confirming that I^-^ is a component in synthesized CuI, 2.5 GA-CuI and 5.0 GA-CuI NPS. The high resolution of the Cu 2p and I 3d spectra show the creation of CuI synthesized both using GA and without using GA, as well as only one oxidation for both Cu and I. Additionally, Figure 2c shows core level spectra for O 1 s orbital peaks in all synthesized CuI NPs, positioned around the 532 eV binding energy, which is assigned to absorbed H_2_O and gallic acid [26].

#### 2.1.3. Field Emission Scanning Electron Microscope (FESEM) Analysis of CuI

**Figure 3:**
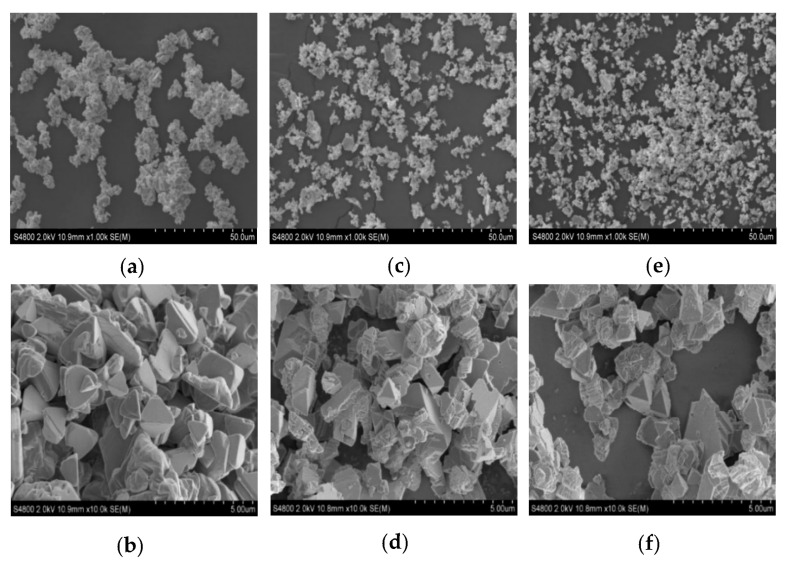
SEM images of CuI NPs with magnifications of (**a**) 1000× (CuI), (**b**) 10,000× (CuI), (**c**) 1000× (2.5 GA-CuI), (**d**) 10,000× (2.5 GA-CuI), (**e**) 1000× (5.0 GA-CuI) and (**f**) 10,000× (5.0 GA-CuI).

The morphology of the synthesized CuI crystals was studied using field emission scanning electron microscopy (FESEM) with a voltage of 2.0 kV. FESEM was performed at 1000× and 10,000× magnification to observe the crystallite shape in various synthesized condition. The results shown in Figure 3 reveal that when gallic acid is not used as a biostabilizer, a triangular shape of copper iodide nanostructures is typically produced. Using 2.5 mM and 5.0 mM of gallic acid produced γ-CuI nanosheets instead of nanoparticles [27]. Triangle-like nanosheets of γ-CuI were grown on each other and generated multi-layer stacks; after gallic acid was added into copper sulfate solution, the copper nanoparticles were generated as a substrate [28]. When potassium iodide was added to the mixture, copper iodide nanosheets were grown on copper substrate. Therefore, using gallic acid as a reducing and stabilizing agent produced triangle-shaped nanosheets. Additionally, focusing on the 1000× magnification, biosynthesis of copper iodide without gallic acid provided more agglomeration of particles than when using gallic acid. This result reveals the important role of gallic acid in particle distribution.

#### 2.1.4. Fourier Transform Infrared Spectrum (FTIR)

**Figure 4:**
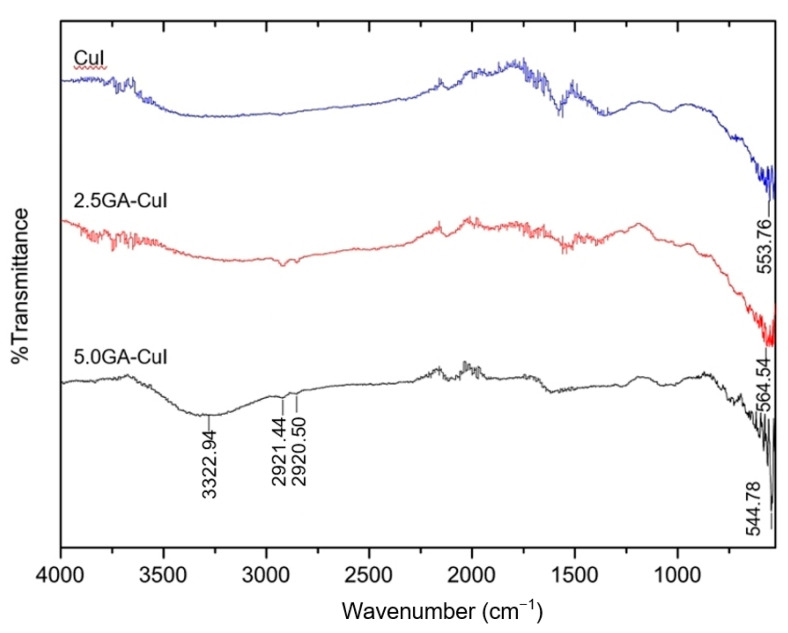
FTIR spectra of green synthesized CuI NPs using different gallic acid (GA) concentrations of 0, 2.5 and 5.0 mM.

Figure 4 compares the FTIR spectra of copper iodide nanoparticles (CuI NPs) synthesized with different contents of gallic acid. There was only one interesting peak at wave number 553.76 cm^−1^ in the spectrum of CuI, related to Cu-I stretching vibrations. The FTIR spectrum of 2.5 GA-CuI only shows important peaks of CuI formation. In the final synthesis, the FTIR spectrum of 5.0 GA-CuI shows a broad band around 3322.94 cm^−1^, assigned to the inter- or intramolecular hydrogen bond (-OH) stretching vibration in gallic acid, which serves as a stabilizer and capping agent of 2.5 GA-CuI. The FTIR peaks at 2921.44 and 2852.29 cm^−1^ represent -CH stretching in gallic acid, while the Cu-I stretching vibration appears in the FTIR peak at a wave number of 544.78 cm^−1^. 

After comparing the FTIR spectra of all synthesized CuI NPs, it can be seen from the spectra that gallic acid serves as a biostabilizer and bioreducer in CuI NP synthesis. Gallic acid at a concentration of 5.0 mM has a greater ability to stabilize CuI than at 2.5 mM, because the higher concentration of gallic acid disassociate in water. In addition, the FTIR peak of 5.0 GA-CuI shows a broad band and a high percent of transmittance at the -OH group. However, 2.5 GA-CuI and CuI NPs did not show any -OH groups in their FTIR spectra. This result is due to the low concentration of GA, which was used for reducing copper ions. GA did not play a biostabilizing role in 2.5 GA-CuI but did play a bioreducing role. Therefore, GA did not attach to CuI and 2.5 GA-CuI, resulting in no -OH groups in their FTIR spectra. The FTIR spectra of all CuI NPs were quite different from those of Ann Candice Fernandez et al. [29], who prepared copper iodide nanoparticles by using cyanidin-3-diglucoside-5-glucoside from red cabbage extract. Their FTIR results for CuI showed an obvious peak of cyanidin-3-fdiglucoside-5-glucoside, which was used in CuI stabilization because cyanidin-3-diglucoside-5-glucoside has a strong hydroxyl group, allowing it to act as a biostabilizer.

#### 2.1.5. Zeta Potential Analysis

The physical properties of synthesized nanoparticles in colloidal systems are generally measured in terms of zeta potential. Zeta potential indicates the dispersion stability and surface properties of molecules or nanoparticles in a liquid medium. Principally, if the magnitude of absolute zeta potential (±ve) is high, molecules or nanoparticles will be stable in a colloidal system because they have high repulsion force, preventing nanoparticles from aggregating [30]. The zeta potentials of the synthesized CuI NPs in different conditions are shown in Table 1. CuI NPs prepared using 0, 2.5 and 5.0 mM gallic acid have average zeta potential values of −13.47, −18.73 and −20.47 mV, respectively. The results show that synthesizing CuI NPs using a greater content of gallic acid leads to greater zeta potential due to the bioreducing properties of gallic acid; thus, these CuI NPs have greater dispersion stability.

#### 2.1.6. Nanosizer/Dynamic Light Scattering

**Figure 5:**
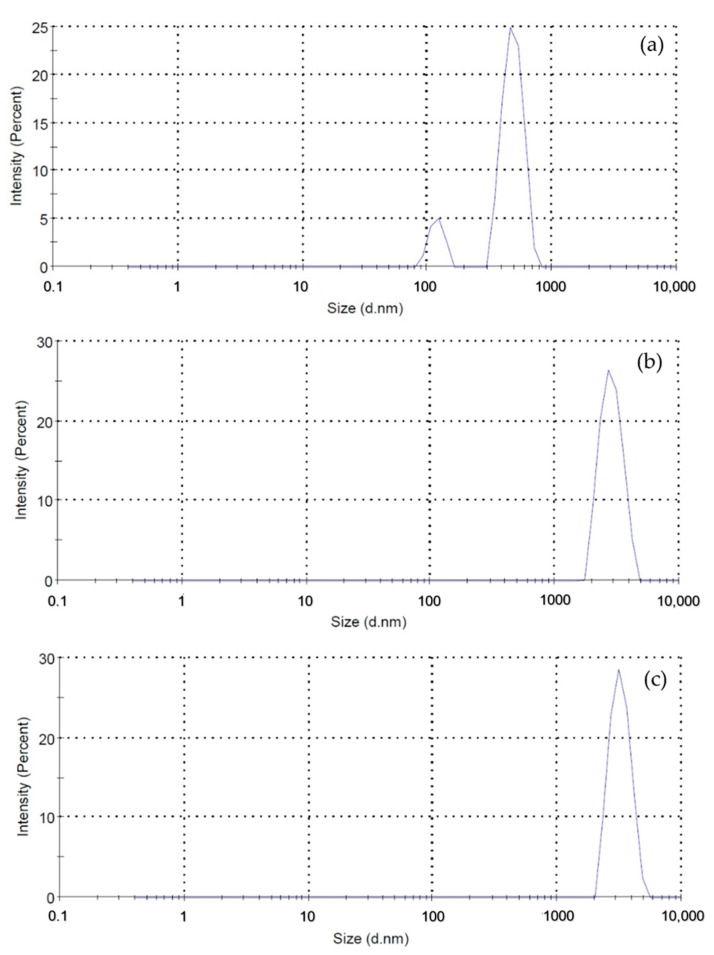
Size distribution of (**a**) CuI NPs, (**b**) 2.5 GA-CuI NPs, and (**c**) 5.0 GA-CuI NPs determined using a nanosizer.

The nano-size of synthesized CuI NPs was measured by using a nanosizer/dynamic light scattering, which illustrates the relationship between intensity and size distribution (nm), as shown in Figure 5. It can be seen from Figure 5a, that the diameter was about 869 nm for CuI NPs synthesized without a biostabilizer. When gallic acid was used to reduce and stabilize CuI NPs, the size distributions of 2.5 GA-CuI and 5.0 GA-CuI were 2473 and 3062 nm, accordingly, as presented in Figure 5b,c. Both 2.5 GA-CuI and 5.0 GA-CuI had greater diameters than CuI NPs. This result reveals that green synthesis of CuI NPs provides electrostatic repulsion from gallic acid molecules, causing larger size distribution [20].

#### 2.1.7. Peroxidase-like Properties of GA-CuI NPs

The therapeutic use of synthesized GA-CuI NPs has been investigated for their enzyme-like properties, which include peroxidase-like activity. This activity is important for the wound healing ability of GA-CuI, because copper nanoparticles function as redox enzymes due to the increased electron exchange of their atoms [31]. The improved antibacterial activity of metal nanoparticles were analyzed in terms of their oxidase-like and peroxidase-like properties, as seen in Equations (1) and (2). When GA-CuI NPs come in contact with bacteria in the presence of oxygen (O_2_), antioxidant chemical (AH_2_) is oxidized and produces hydrogen peroxide (H_2_O_2_). Then, H_2_O_2_ is further catalyzed by the peroxidase-like enzymes of GA-CuI NPs, generating hydroxyl radicals (•OH). Hydroxyl radicals (•OH) are important molecules for killing bacteria by interacting with the bacterial cell wall and oxidizing biomolecule. Therefore, the oxidase- and peroxidase-like activity of GA-CuI NPs gives them a strong antibacterial capacity through the mechanisms of hydrogen peroxide and hydroxyl radical production.

(1) $$AH_{2}\;+\;O_{2}\;\to \;A\;+\;H_{2}O_{2}$$

(2) $$H_{2}O_{2}\;\overset{GA\;-\;CuI\;NPs}{\to }\;2\cdot OH$$

**Figure 6:**
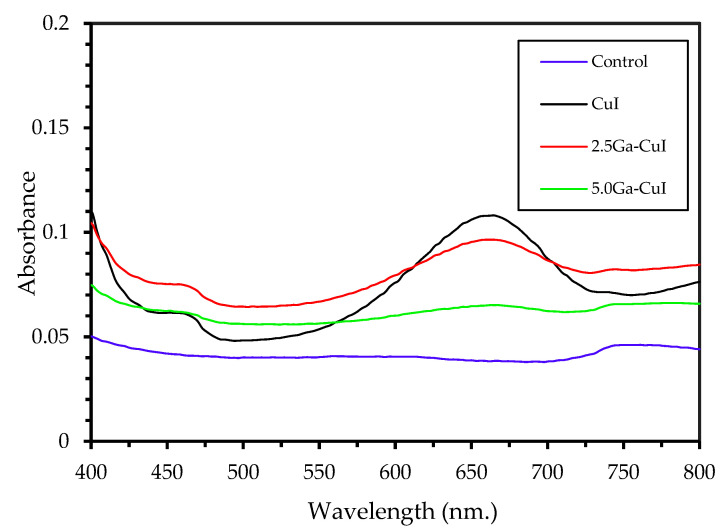
UV–Vis absorption spectra of 3,3′,5,5′-tetramethyl-benzidine (TMB) oxidation, treated with acetate buffer (light blue), CuI (black), 2.5 GA-CuI (red) and 5.0 GA-CuI (green).

In the investigation of peroxidase-like activity, 3,3′,5,5′-tetramethyl-benzidine (TMB) was used as a substrate for peroxidase. Notice the color change after treatment with CuI, 2.5 GA-CuI and 5.0 GA-CuI NPs. A UV–Vis spectrometer was employed to observe the absorbance peaks of oxidized TMB (TMB^+^). It can be seen from Figure 6 that the absorbance peaks of TMB treated with CuI, 2.5 GA-CuI and 5.0 GA-CuI were detected around 650 nm. TMB oxidation peaks appeared after treatment with CuI and 2.5 GA-CuI, while 5.0 GA-CuI exhibited a weak oxidizing effect on TMB. This may be due to the decreased amount of CuI when concentrations of gallic acid are increased. Consequently, the capacity of 5.0 GA-CuI to oxidize TMB is poor. However, the weight loss of CuI, 2.5 GA-CuI and 5.0 GA-CuI must be examined to determine the amount of each chemical in the compound.

#### 2.1.8. Evaluation of Minimum Inhibitory Concentration (MIC) and Minimum Bactericidal Concentration (MBC) of CuI NPs

**Figure 7:**
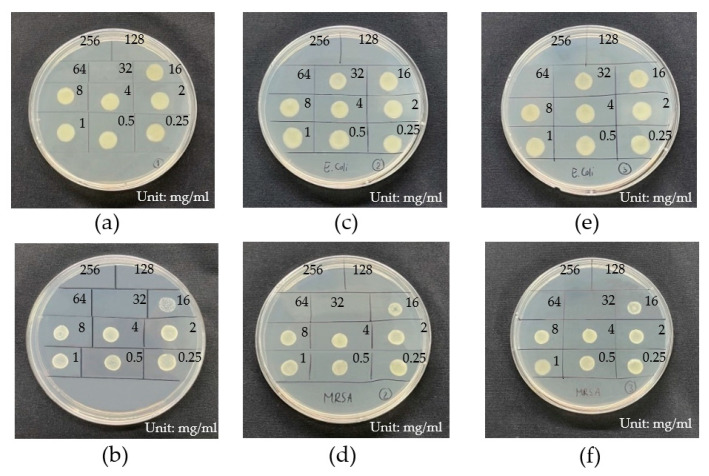
Bacterial growth after treatment with different synthesized CuI NPs for each bacterial strains: (**a**) *E. coli* treated with CuI; (**b**) MRSA treated with CuI; (**c**) *E. coli* treated with 2.5 GA-CuI; (**d**) MRSA treated with 2.5 GA-CuI; (**e**) *E. coli* treated with 5.0 GA-CuI; (**f**) MRSA treated with 5.0 GA-CuI.

**Figure 8:**
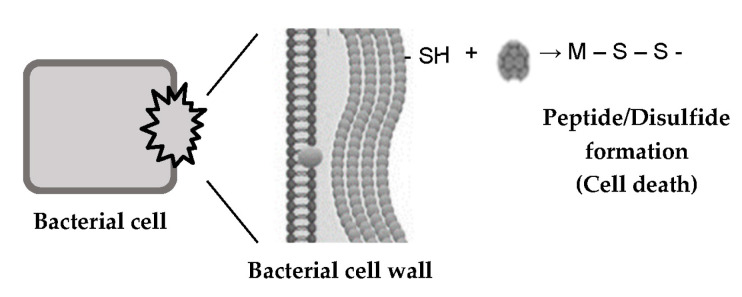
Antibacterial mechanism of CuI NPs through denaturation.

The antibacterial activity of synthesized CuI NPs was tested against *E. coli* (Gram- negative bacteria) and MRSA (Gram-positive bacteria), as these are the most common bacterial infections in wounds and the bloodstream. The inhibitory effect of CuI NPs on bacterial growth was studied with various concentration of CuI NPs, using the broth serial dilution method to evaluate minimum inhibitory concentration (MIC) and minimum bactericidal concentration (MBC). Figure 7 illustrates the results of bacterial growth on agar plates; these *E. coli* and MRSA were treated with different concentrations of synthesized CuI NPs. The CuI NPs were diluted to 256, 128, 64, 32, 16, 8, 4, 2, 1 and 0.5 mg/mL, represented by numbers 1, 2, 3, 4, 5, 6, 7, 8, 9 and 10, respectively. In addition, Figure 7a,b show bacterial growth after treatment with CuI synthesized without gallic acid. Figure 7c,d present bacterial growth after treatment with 2.5 GA-CuI. Bacterial growth after treatment with 5.0 GA-CuI is shown in Figure 7e,f. It can be seen from Table 2, which shows the MIC and MBC values of different CuI NPs for *E. coli* and MRSA strains, that CuI at 32 mg/mL completely inhibited both Gram-negative (*E. coli*) and Gram-positive (MRSA) bacteria, whereas 2.5 GA-CuI and 5.0 GA-CuI eliminated Gram-negative bacteria (*E. coli*) at a higher concentration of 64 mg/mL. Therefore, it can be concluded that CuI has a greater antibacterial effect than 2.5 GA-CuI and 5.0 GA-CuI, because 2.5 GA-CuI and 5.0 GA-CuI were composed of gallic acid and copper iodide. The antibacterial mechanism of CuI occurs through the denaturation of solid-state compounds, which deforms the bacterial cell wall. Synthesized CuI NPs have a high potential to bind with the thiol group of proteins in the cell wall, forming peptide/disulfide complexes. This activity can cause cell death, as illustrated in Figure 8 [32].

The proportion of pure CuI NPs in 2.5 GA-CuI and 5.0 GA-CuI is lower than in CuI, and CuI is the main bactericidal factor. Additionally, CuI synthesized without GA is smaller in size than with GA. The bacteria-killing ability of CuI NPs is also dependent on particle size, as CuI NPs of smaller size have more potential to damage bacterial cells than those of larger size. This is because smaller CuI NPs have greater ability to enter the cell wall and create physical attachments deforming it. Therefore, CuI NPs are more efficient at killing bacteria [16].

In addition, Gram-negative bacteria (*E. coli*) are more resistant to CuI NPs than Gram-positive bacteria (MRSA). This could be because *E. coli* has a more complex structure and because the outer membrane of Gram-negative bacteria acts as a barrier, providing additional protection against CuI NPs.

### 2.2. PVA/CuI Liquid Bandage Formulation

#### 2.2.1. Fourier Transform Infrared Spectroscopy (FTIR)

**Figure 9:**
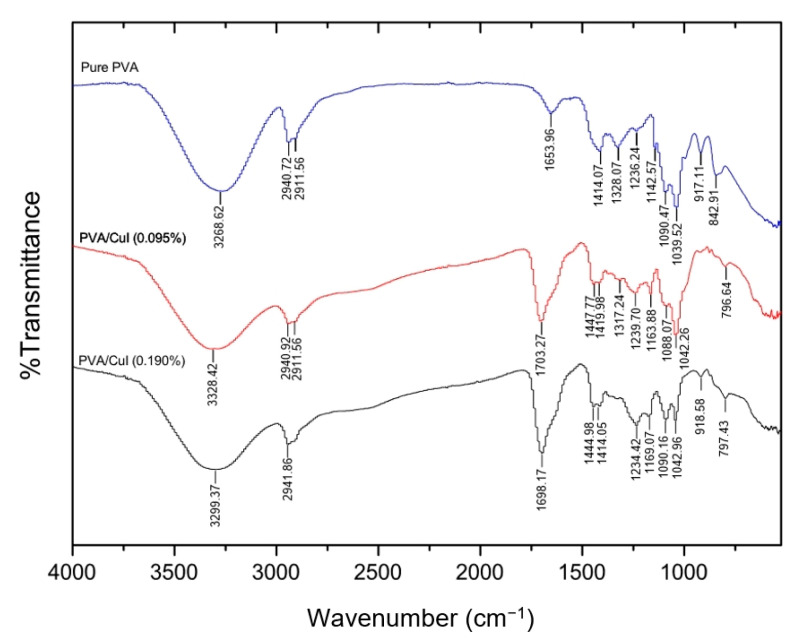
FTIR spectra of PVA and PVA incorporating CuI suspension.

Figure 9 illustrates the FTIR spectra of pure PVA and PVA with different concentrations of CuI suspension (0.095% and 0.190%). The characteristic peaks of PVA were observed at 3268.62, 2940.72, 2911.56, 1653.96, 1414.07, 1328.07, 1236.24, 1090.47, 1039.52 and 842.91 cm^−1^. These peaks were designated as -OH stretching, asymmetric stretching of CH_2_, symmetric stretching of CH_2_, C=O carbonyl stretching, -CH_2_ bending, C-H wagging, C-OH stretching in alcohol, C-O stretching, C=O stretching and bending of -OH and C-C stretching, respectively. In the PVA solution with 0.095% CuI, peaks were noticed at wave numbers 3328.42, 2940.72, 2911.56, 1703.27, 1447.77, 1317.24, 1239.70, 1088.07, 1042.26 and 796.64 cm^−1^. These peaks were assigned to characteristic PVA peaks with a minor shift, comparable with pure PVA [33]. Similarly, important peaks of PVA were observed in PVA with 0.190% CuI. The vibration spectra of the materials are presented in Table 3. The C–H scissoring vibration mode is predicted at about 1440 cm^−1^ for pure PVA. In this experiment, the C–H scissoring vibration was clearly observed only after the addition of CuI NPs to the PVA solution, which causes a structural deformation of the PVA backbone. In addition, for pure PVA, the vibration pattern of carbonyl stretching is expected at about 1650 cm^−1^, which is the result of residual acetate in the polymerization. The carbonyl (C=O) stretching vibration was found at 1703.27 cm^−1^ and 1698.17 cm^−1^ for PVA/CuI (0.095%) and PVA/CuI (0.190%), respectively. It is clear that the transmittance of the carbonyl stretching was increased in PVA with CuI NPs, as they increase the presence of carbonyl moieties. This is due to the addition of CuI complexes, while polymerizing PVA can cause the breaking of –H and –OH bonds and, as a result, the formation of more carbonyl double bonds (C=O). Accordingly, the intensity of the C=O stretching peaks was increased and shifted to higher wavelengths. Furthermore, the characteristic peak of CuI NPs was not present in the FTIR spectra of PVA/CuI (0.095%) and PVA/CuI (0.190%) because this characteristic peak is lower than 525 cm^−1^, while 525 cm^−1^ is the minimum wave number for this FTIR spectroscopy. However, PVA with CuI NPs in its structure can be confirmed from intensity and shift of the wave numbers. 

#### 2.2.2. Surface Morphology Analysis of Cast PVA Film Using Field Emission Scanning Electron Microscopy (FESEM)

**Figure 10:**
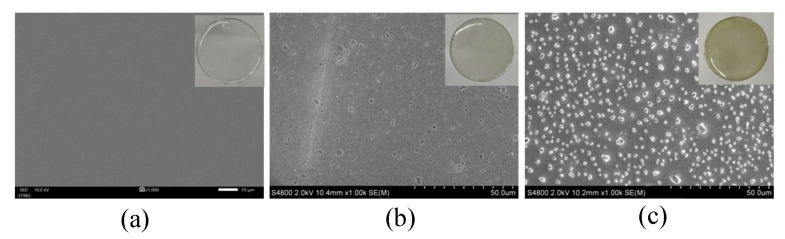
SEM image of cast PVA film: (**a**) pure PVA, (**b**) PVA/CuI (0.095%) and (**c**) PVA/CuI (0.190%).

Figure 10 presents SEM images of the poly(vinyl alcohol) nanocomposite material after the incorporation of copper iodide nanoparticles (5.0 GA-CuI) in the polymer matrix prepared by cast film method. The magnification used to study the morphological surface was 1000×. The surface morphology of PVA film with 0, 0.095% and 0.190% CuI suspension is shown in Figure 10. Figure 10a shows the smooth surface observed on pure PVA film. After 0.095% copper iodide nanosheets were loaded onto PVA film, there were multi-sized granular particles on the surface of the composite. This shows the good distribution of smaller non-agglomerated particles. Then, more CuI suspension (0.190%) was added to the PVA solution. The morphology of the surface demonstrated that CuI NPs were excellently distributed in PVA film. In spite of the high concentration of copper iodide particles, excellent distribution was achieved due to GA-CuI stability. 

#### 2.2.3. Swelling Behavior of Pure PVA and PVA/CuI Film

The swelling ratio is used to determine the resistance of polymer film when exposed to high humidity or water in the bloodstream or at the wound site [34]. Tests of the swelling behavior of PVA film were conducted using gravimetric measurement [35]. The average thicknesses of pure PVA, PVA/CuI (0.095%) and PVA/CuI (0.190%) were 0.53, 0.59 and 0.60 mm, respectively. All PVA films were immersed in PBS buffer solution at 37 °C to provide a constant pH of 7.2 ± 0.1. After the specific swelling time, each film was removed from the PBS solution, and surface water was rapidly cleaned using Kimwipes paper. Then, each sample was weighed and recorded with an electronic balance. The swelling ratio of PVA films was calculated.

**Figure 11:**
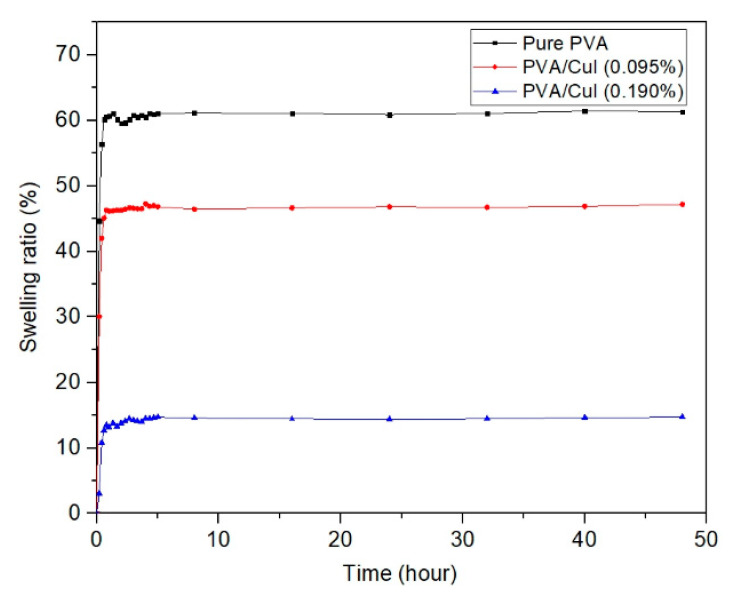
The swelling behavior of pure PVA, PVA/CuI (0.095%) and PVA/CuI (0.190%).

Figure 11 illustrates the swelling behavior of pure PVA, PVA/CuI (0.095%) and PVA/CuI (0.190%), shown by the black, red and blue lines, respectively. An increase in the swelling ratio of all PVA films over time was noticed; the swelling rate considerably increased at the initial time and then leveled off. The red and blue lines show the incorporation of CuI NPs in PVA films, which significantly reduced the swelling behavior. The swelling ratio decreased with the increasing load of CuI NPs. This is due to the hydrophobic nature of CuI NPs. The more CuI NPs are loaded in PVA films, the greater the repulsion of water molecules. Therefore, CuI NPs loaded in PVA liquid bandages enhance resistance to humidity and blood after they are cast to thin film.

#### 2.2.4. Drying Time of PVA Liquid Bandages

**Figure 12:**
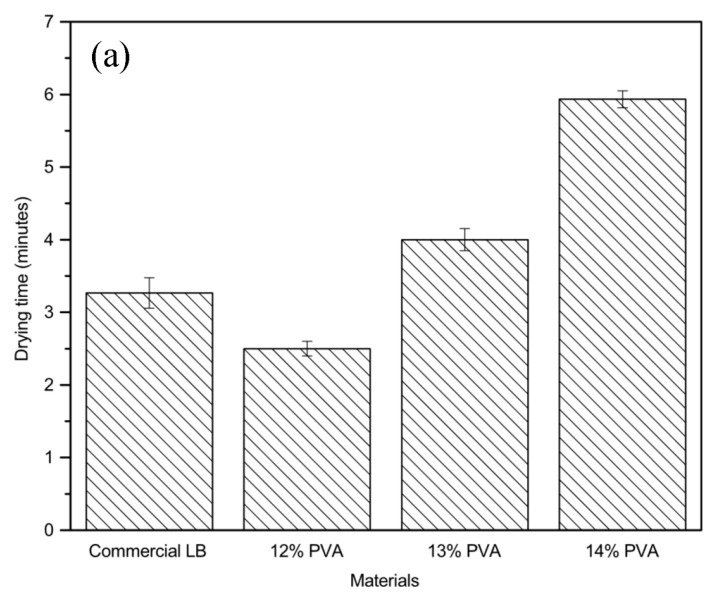

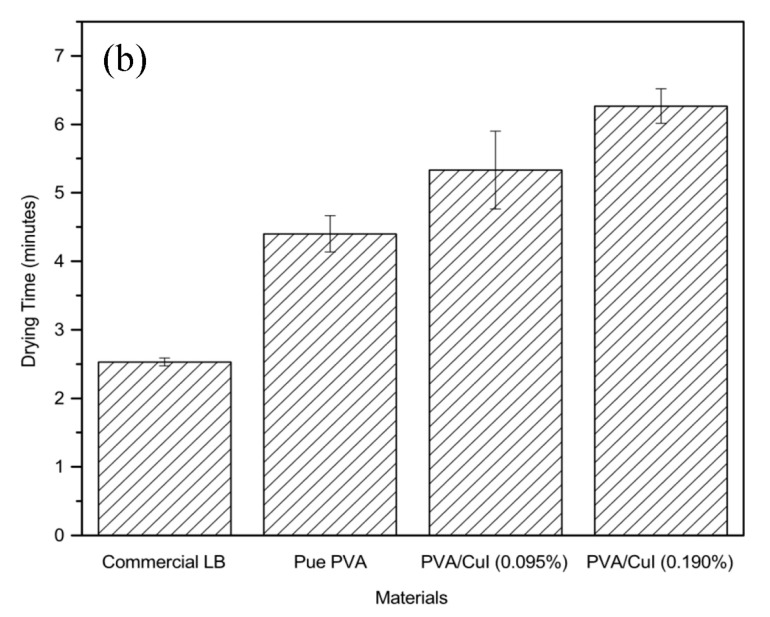
Drying time of film compared with a commercial liquid bandage (LB): (**a**) content of PVA; (**b**) PVA composed of CuI suspension.

The drying time of liquid bandage formulations synthesized with different contents of poly(vinyl alcohol) was tested by applying 0.2 mL of 12%, 13% and 14% PVA liquid solution on the inside forearm of a volunteer and letting it dry. The drying time and physical properties of each PVA concentration were recorded after there was no visible PVA liquid on the skin. The drying time and physical properties of the liquid bandages are shown in Figure 12. The drying time of 12%, 13% and 14% PVA liquid bandages were 2.5, 4.0 and 6.0 min, respectively. Even though 12% PVA provided the minimum drying time, the PVA content was not enough for it to spread as a matrix, as shown in Figure 12a. Additionally, 14% PVA content showed significantly increased drying time due to the greater amount of PVA, which has high viscosity and requires more time for particles to coalesce and form a film. Therefore, the most suitable concentration of PVA is 13%.

After the appropriate PVA content was determined, the drying times of different PVA liquid bandages loaded with CuI suspension were subsequently examined. PVA solution incorporating different concentrations of CuI suspension (0, 0.095% and 0.190%) were prepared and drying time was tested. It can be seen from Figure 12b that the drying time increased with increasing concentrations of CuI suspension, showing that higher contents of CuI result in longer drying times, because a large amount of CuI NPs provides high viscosity and a difficult pathway for evaporation of the cosolvent [36].

#### 2.2.5. Water Vapor Transmission Rate (WVTR)

**Figure 13:**
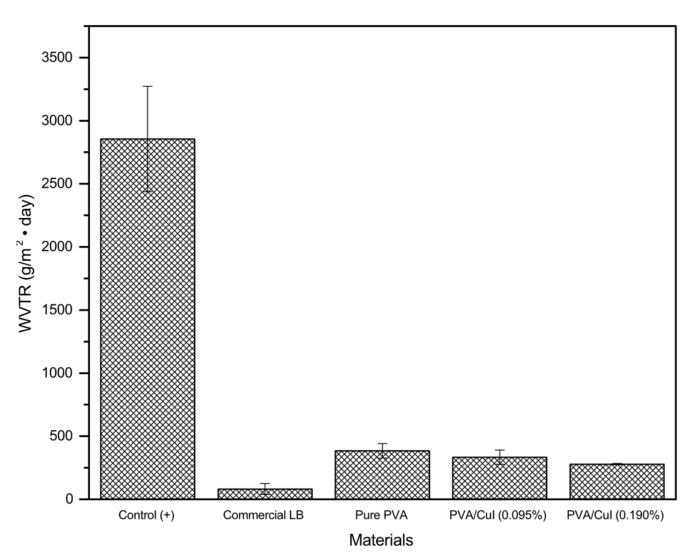
Water vapor transmission rate (WVTR) for each synthesized liquid bandage.

It is necessary to study the water vapor transmission rate because this property helps promotes wound healing and contraction of the wound. Bleeding will occur from superficial wounds. Water is the most important component of blood (90%); therefore, a good water vapor transmission rate allows blood to exit the wound dressing and enhances wound drying. The water vapor transmission rate (WVTR) of the PVA samples was tested under virtual conditions, compared with a centrifuged tube without any cover as a control (+), as shown in Figure 13. The WVTRs of pure PVA, PVA/CuI (0.095%), PVA/CuI (0.190%) and a commercial liquid bandage (LB) were 384.33, 333.64, 278.23 and 81.35 g/m^2^ • day, respectively. It can be seen that the water vapor transmission rate (WVTR) decreases with increasing concentrations of CuI due to the decreased diffusion rate of water vapor through the PVA composite film [37]. Compared with commercial liquid bandage, PVA film has a higher WVTR. Therefore, PVA film has good ability to allow water to evaporate from wounds, promoting rapid wound healing [38].

#### 2.2.6. In Vitro Cytotoxicity and Biocompatibility of Liquid Bandage

**Figure 14:**
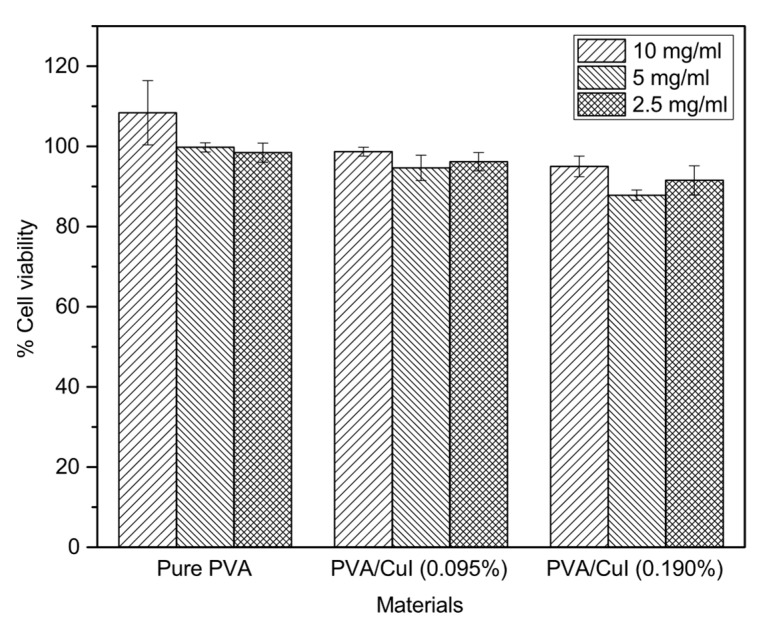
Surviving cells after treatment with each sample extraction.

In vitro cytotoxicity was tested to determine the cytotoxicity of various concentrations of CuI suspension in PVA liquid bandages. In this method, the ratio of cell viability of human dermal fibroblasts (HDFa) after 24 h incubation in sample extractions at different concentrations (2.5, 5 and 10 mg/mL) is measured, and exposure to the various liquid bandage was used as an indicator of whether these substances can cause damage to human cells [39]. Figure 14 shows the results, revealing that after HDFa was incubated in sample extractions for 24 h, cell viabilities with 2.5, 5 and 10 mg/mL pure PVA were 108.363, 99.7611 and 98.447%, respectively. HDFa incubated in 2.5, 5 and 10 mg/mL PVA/CuI (0.095%) showed cell viabilities of 98.685, 94.624 and 96.177%, respectively. HDFa incubated in 2.5, 5 and 10 mg/mL PVA/CuI (0.190%) showed cell viabilities of 94.982, 87.814 and 91.517%. The results reveal that total PVA liquid bandages were non-cytotoxic to HDFa at the concentrations tested. Nevertheless, the number of dead cells increased with increasing content of CuI suspension. The increase in cytotoxicity may show that high concentrations of CuI metallic complexes can cause cell damage. However, PVA liquid bandages with CuI suspension demonstrated biocompatibility, allowing them to be used for antibacterial wound dressing.

#### 2.2.7. Antibacterial Efficacy Testing by Time–Kill Assay

**Figure 15:**
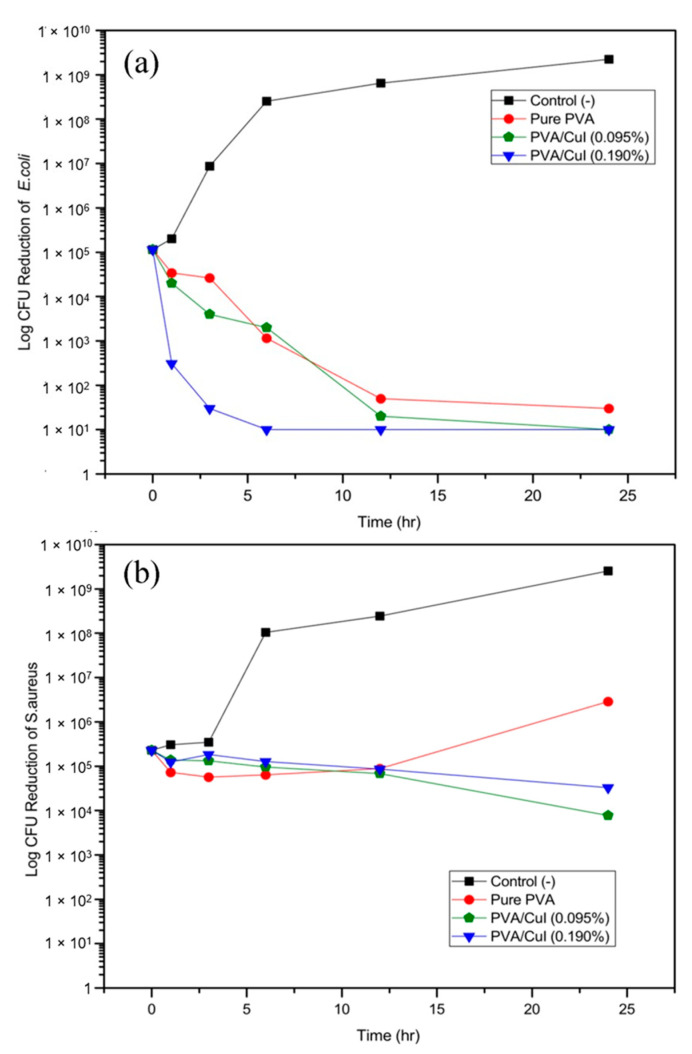
Bacterial reduction of *E. coli* (**a**) and MRSA (**b**) after incubation in liquid bandage.

The time–kill assay was employed to test antibacterial efficacy, exploring the rate of bacterial reduction after being incubated in different concentrations of materials for 1, 3, 6, 12 and 24 h. In vitro antibacterial tests were conducted against Escherichia coli (*E. coli* ATCC25922) and methicillin-resistant *Staphylococcus aureus* (MRSA), which are Gram-positive and Gram-negative pathogens, respectively. Figure 15 shows the rate of bacterial reduction, with the concentration-dependent and time-dependent bactericidal activities of pure PVA, PVA/CuI (0.095%) and PVA/CuI (0.190%) against *E. coli* (a) and MRSA (b). 

Regarding the ability of the materials to kill Gram-negative bacteria (*E. coli* ATCC2592) measured in the rate of log CFU reduction and surviving bacteria counted, it can be observed from Figure 15a that there was a decline in the number of bacterial cells after treatment with pure PVA, PVA/CuI (0.095%) and PVA/CuI (0.190%). Focusing on the bactericidal properties of the pure PVA bandage solution, even though there was no CuI suspension in the PVA solution, a reduction of *E. coli* was noticed because ethanol was added into the mixture as a cosolvent with water. Therefore, ethanol can play an important role in killing bacteria. PVA/CuI (0.095%) and PVA/CuI (0.190%) were able to effectively kill the bacteria. PVA/CuI (0.190%), in particular, was toxic to *E. coli* after only 6 h of incubation due to its high concentration of CuI. PVA/CuI (0.095%) eliminated *E. coli* after treatment for 24 h. The bacterial reduction corresponded to the amount of CuI suspension in the PVA solution. 

Regarding bactericidal properties against MRSA (Figure 15b), the direction of the log CFU reduction for MRSA was completely different from that for *E. coli*. PVA solution without CuI suspension when used in bandages has relatively low capability to inhibit bacterial growth. However, bacterial cell count was reduced when compared with the control (-) due to ethanol. When 0.095% and 0.190% CuI was included in the PVA solution, the trend of cell reduction marginally declined; thus, these materials were not toxic to MRSA. The difference in bacterial reduction for *E. coli* and MRSA is related to the differing bacterial cell structures of Gram-positive and Gram-negative bacteria. The presence of CuI NPs did not change the viability of MRSA, most likely because of its complex structure. These bacteria are strongly resistant to antibiotic drugs. Some strains can develop high-level resistance and pose a serious threat.

The overall results reveal that PVA liquid bandages incorporating CuI suspension can damage both Gram-positive and Gram-negative bacteria, as can be seen from decline of log CFU reduction. However, the antibacterial activities of the materials against *E. coli* were greater than against MRSA.

## 3. Conclusions

In conclusion, antibacterial liquid bandages combining the biocompatible polymer poly(vinyl alcohol) and the antibacterial agent copper iodide, synthesized using gallic acid as a bioreducer and biostabilizer, were effectively designed and characterized. The XRD results of CuI synthesized with gallic acid confirm the crystallinity of synthesized CuI in the γ-CuI phase. As calculated with Scherrer’s equation, the nanoparticle size of copper iodide increased with increasing concentrations of gallic acid. The stability of synthesized copper iodide nanoparticles (CuI NPs) was confirmed by zeta potential analysis. This result agreed with the FTIR spectra of synthesized CuI NPs, which showed that more gallic acid creates more chelation in CuI NPs, seen in the -OH stretching peak, thus leading to high particle stability. The curative effects of synthesized CuI loaded in PVA solution to formulate a liquid bandage were characterized in terms of antibacterial activities against *E. coli* and MRSA, and cytotoxicity to human dermal fibroblasts (HDFa). The antibacterial test (time–kill assay) showed that the synthesized liquid bandages can successfully kill *E. coli* within 6 h and cause a reduction in MRSA. In addition, PVA liquid bandages are non-toxic to HDFa cells with CuI loads of 0.095% and 0.190% *w*/*v*.

## 4. Materials and Methods

### 4.1. Materials

Gallic acid (GA, 97.5–102.5% titration) with molecular weight of 170.12 g/mol was purchased from Sigma-Aldrich, St. Louis, MO, USA. PVA with a molecular weight of 89,000–90,000 g/moL, copper sulfate tetrahydrate (CuSO_4_ • 5H_2_O) and potassium iodide (KI) were purchased from Sigma-Aldrich, USA.

### 4.2. Preparation of Gallic Acid–Cuprous Iodide Nanoparticles

The cuprous iodide nanoparticles stabilized by gallic acid (GA-CuI NPs) were prepared using different concentrations of gallic acid (0, 2.5 and 5.0 mM). In the case of cuprous iodide without gallic acid, 1.2400 g. of CuSO_4_ • 5H_2_O was dissolved in 50 mL deionized water in a 100 mL volumetric flask to make 50 mM of copper sulfate solution. Then, 50 mM of potassium iodide was prepared. Potassium iodide (KI) in the amount of 0.8300 g was dissolved in 50 mL deionized water in a 100 mL volumetric flask, and the concentration was adjusted. After, potassium iodide was added drop-wise to the copper sulfate solution with continuous stirring for 45 min. Then, the solution was centrifuged at 6000 rpm for 20 min, washed with deionized water to remove unreacted substances and centrifuged again. Finally, the precipitation was collected and dried in an oven at 60 °C for 6 h.

For the varying gallic acid concentrations, 0.0123 and 0.0245 g of gallic acid was dissolved in 30 mL deionized water to prepare 2.5 mM and 5.0 mM of gallic acid, respectively. Then, a constant amount of copper sulfate (50 mM) and potassium iodide (50 mM) were prepared similarly to the previous step. After, a varying amount of gallic acid (2.5 mM and 5.0 mM) was added drop-wise to the copper sulfate solution in 250 mL beakers with constant stirring for 15 min, allowing the gallic acid to stabilize and reduce the Cu^2+^ to Cu^+^. A color change was observed. Each beaker of potassium iodide was added drop-wise to the mixture and stirred for 45 min. The solution was centrifuged at 6000 rpm for 20 min, washed by ethanol and deionized water to remove unreacted substances and centrifuged again. Finally, the precipitation was collected and dried in an oven at 60 °C for 6 h. 

### 4.3. Preparation of PVA Antibacterial Liquid Dressing with Additional Gallic Acid–Cuprous Iodide 

After the most suitable CuI NP solution was selected, copper iodide was loaded into the adhesive bandage formulation, in which PVA was used as a film former and glycerol as a softening agent. This creates a kind of liquid adhesive bandage. Calculated by weight percentage, 13% *w*/*v* of PVA was dissolved in 40% ethanol in water. Then, the mixture was heated to 75 °C, allowing the film former to fully dissolve. After, 6% *w*/*v* of glycerol softening agent was added to the PVA solution under continuous stirring, 0, 0.095% and 0.190% *w*/*v* of selected CuI NP antibacterial agent was separately added to the PVA solution and stirred for 15 min. Finally, the antibacterial PVA liquid bandage solutions were obtained.

### 4.4. Crystal Structure of CuI NPs

The crystal structure of the synthesized CuI, 2.5 GA-CuI and 5.0 GA-CuI NPs was characterized by X-ray diffraction (Rigaku SmartLab X-ray diffractometer), using standard XRD measurements with a wavelength of 1.54 Å Cu Kα radiation. The X-ray diffractometer was applied at a voltage of 40 kV and a current of 30 mA. All samples were scanned with a diffraction angle of 2θ from 20 to 80 degrees, with a scanning rate of 0.02 degrees per second. 

### 4.5. Characterization of Microstructure and Morphology of Synthesized CuI NPs and PVA Liquid Bandage

Scanning electron microscopy (SEM; JEOL JSM-6610LV, Oxford X-Max 50, Tokyo, Japan) was used to investigate the microstructure, morphology and average size of gallic acid–CuI nanoparticles. Different conditions of CuI synthesis were examined at 500× magnification at 300 kV. In addition, SEM was used to investigate the morphology of the pure PVA film surface and PVA with CuI solution. The samples were coated with platinum using a sputtering device prior to SEM observation. 

### 4.6. Functional Group Determination of CuI NPs and PVA Liquid Bandage 

Fourier transform infrared spectroscopy (FTIR; Nicolet iS 5, Massachusetts, United States) was used to determine the functional group containing gallic acid–CuI NPs. Scans were conducted from 4000 to 600 cm^−1^ with the iD1 Transmission module, with the FTIR spectrum scanned 64 times. For characterization of PVA liquid bandage, the PVA liquid bandage with and without CuI solution was also analyzed with Fourier transform infrared spectroscopy (FTIR; Nicolet iS5). 

X-ray photoelectron spectroscopy (XPS) analysis (Kratos, Axis ultra DLD, New York, United States) was employed to investigate the exact chemical state (oxidant state) of the elements composing the synthesized compounds. 

### 4.7. Size Determination of CuI NPs

A zeta potential analyzer (MALVERN Zetasizer Nano ZSP, Worcestershire, England) was used to determine the stability and surface properties of CuI nanoparticles synthesized with different amounts of gallic acid in deionized water. In addition, a nanosizer was utilized to measure the size distribution of synthesized CuI NPs. 

### 4.8. Peroxidase-like Activity of CuI NPs

The protocol for measuring peroxidase-like properties was taken from Li Wang et al. [18] with some modifications. A UV–Vis spectrophotometer was used to determine the peroxide-like reaction. Additionally, 3,3′,5,5′-tetramethyl-benzidine (TMB) was used as a substrate to examine the peroxidase-like property of CuI and GA-CuI NPs. An acetate buffer (pH = 5.5) was used as a control. There were four groups in the peroxidase test, including TMB + H_2_O_2_ + acetate buffer (control), TMB + CuI NPs + H_2_O_2_, TMB + 2.5 GA-CuI NPs + H_2_O_2_ and TMB + 5.0 GA-CuI NPs + H_2_O_2_. The peroxidase-like property of CuI NPs was investigated at a pH condition of 5.5 in acetate buffer, because these are the conditions during bacterial infection after a wound has occurred. In detail, TMB was dissolved in DMSO to prepare 1 mM of TMB solution; then, 150 μg of each sample (CuI, 2.5 GA-CuI and 5.0 GA-CuI NPs) was separately prepared in 1 mL of acetate buffer. Finally, 1 mM of H_2_O_2_ was prepared in deionized water. After the required solutions were completely prepared, 20 μL of TMB, 200 μL of each sample and 180 μL of H_2_O_2_ were mixed together in each group and kept in dark conditions for 10 min. The absorbance of TMB treated with CS-Cu-GA NCs at 650 nm was measured using a UV–Vis spectrometer. 

### 4.9. Evaluation of Minimum Inhibitory Concentration (MIC) and Minimum Bactericidal Concentration (MBC) of CuI NPs

The antibacterial activity of gallic acid–CuI NPs against Gram-negative *E. coli* (ATCC 25922) and Gram-positive methicillin-resistant *Staphylococcus aureus* (MRSA, ATCC 33591) was tested by broth dilution method to evaluate minimum inhibitory concentration (MIC) and minimum bactericidal concentration (MBC). Gram-negative *E. coli* and Gram-positive MRSA were separately dispersed in saline solution; the optical density was measured to be 0.5 using a densitometer, resulting in 1.5 × 10^8^ CFU/mL of bacteria. Test tubes containing 1.5 × 10^8^ CFU/mL were diluted by pipetting 100 µL into 3 mL of saline solution; then, 5 × 10^6^ CFU/mL of *E. coli* and of MRSA was obtained. After the bacterial suspension was prepared, serial dilution of the copper iodide solution was conducted. An amount of 512 mg of copper iodide was dissolved in 2 mL of DMSO, and 2 mL of MHB was added to this test tube. Then, serial dilution was conducted 10 times in 24-well plate. Bacterial suspension of 5 × 10^6^ was transferred to a 24-well plate from column 1 to column 10 and was incubated in a shaking incubator with overnight cultures of bacteria.

### 4.10. Physical Properties of PVA Liquid Bandage Formulation

To evaluate the drying time of the PVA formulations on human skin, a test was applied on the inner side of the forearm. Different PVA formulations were spread on human forearm and let dry for a period of 1, 2 and 3 min. If there was no liquid visible on the skin, the PVA film was considered to be dry. The PVA liquid bandage with minimum drying time was chosen to create a liquid bandage.

Additionally, the water vapor transmission rate describes the amount of water passed through a unit area of film in a unit time. The water vapor transmission rate was evaluated to study the water permeability of the films, as this has an effect on skin temperature and the hydration of the wound. Following Zurdo Schroeder et al. [40], PVA liquid bandages loaded with different amounts of CuI suspension were cast on glass plates to evaporate the solvent, forming PVA films. These films were cut into circular samples with diameters of 0.03 m and covered on centrifuge tubes containing 30 mL of distilled water. The tubes were placed into an incubator with a temperature of 37 °C and relative humidity of 52%. Then, the tubes were weighed at each time point—0, 3, 6 and 24 h—to calculate the water vapor transmission rate. The weight loss of the glass tubes was determined as ∆m (g), m_0 h_–m_24 h_. The water vapor transmission rate is calculated by the amount of water transmitted through in PVA film related to surface area A (m^2^) and time t (day), following the equation below:

$$\mathrm{Water}\;\mathrm{vapor}\;\mathrm{transmission}\;\mathrm{rate}\;(\mathrm{WVTR})=\frac{\Delta m\;(g)}{{A\;(m}^{2})\;\times \;\mathrm{time}\;(\mathrm{day})}$$

The swelling ratio is usually measured for polymer hydrogel or polymer films to determine water resistance. The study of swelling behavior was performed referring to J. Liu et al. [36]. Pure PVA, PVA/CuI (0.095%) and PVA/CuI (0.190%) were cast in glass Petri dishes to form films. Then, each film was cut into a circular film with a diameter of 12 mm, and the average thickness was measured. After that, PBS buffer solution was prepared with sodium chloride, potassium chloride, sodium phosphate dibasic and monopotassium phosphate to make a buffer solution with pH 7.2 ± 0.1. Each film was weighed before the swelling test; the weight was determined as m_0_. Then, the samples were incubated at 37 °C. After the specified time, the samples were removed from PBS, then quickly cleaned with Kimwipes paper to remove water and weighed with an electronic balance. At time t, the weight was recorded as m_t_. All samples were continuously incubated in PBS solution and weighed at each time point up to 48 h to ensure that equilibrium was reached. The swelling ratio was calculated by the equation below:

$$\mathrm{Swelling}\;\mathrm{ratio}=\frac{m_{t\;}{-\;m}_{0}}{m_{0}}\;\times \;100$$

### 4.11. In Vitro Indirect Cytotoxic Test of PVA Liquid Bandage Formulations

The indirect cytotoxicity of PVA loaded with CuI suspension was tested following the ISO10993-5 (biological evaluation of medical devices) standard test using adult human dermal fibroblasts (GIBCO, Grand Island, NY, USA). Firstly, the culture medium was prepared and collected by exposing samples to UV radiation for 30 min for sterilization. Then, the samples were immersed in DMEM in a 96-well tissue culture polystyrene (TCPS) plate at 0.5, 5 and 10 mg/mL for 1 and 3 days to produce different concentrations of sample extractions. After, the samples were placed into a 24-well culture plate containing of Dulbecco’s phosphate-buffered saline modified with Eagle’s medium (Hyclone, Logan, UT, USA), with the addition of 10% fetal bovine serum (Gibco), 1% L-glutamine (Gibco), 100 µg/mL of streptomycin, 100 units/mL of penicillin (Gibco) and 5 µg/mL of amphotericin B (Gibco) for 24 h. Then, 10,000 cells of the human dermal fibroblast (HDFa) cell line were cultured in the prepared medium in a 96-well plate and incubated overnight in a humidity chamber with 5% CO_2_ at 37 °C. The number of viable cells were counted using (4,5-dimethylthiazol-2-yl)-2,5-diphenyl-tetrazolium bromide (MTT) assay (USB Corporation, Cleveland, OH, USA). An absorbance microplate reader (BiotekELx800; Biotek Instruments Inc., Winooski, VT, USA) was used to determine the optical density (O.D.) at 570 nm of cells. The control group was 2D cell culture in normal conditions.

### 4.12. Antibacterial Test by Time–Kill Assay

The antibacterial activities of PVA liquid bandage with different concentration of CuI suspension were characterized through time–kill assay. It was necessary to determine the ability of the liquid bandage to kill bacteria in a fixed time. Briefly, the antibacterial activity of PVA liquid solutions was tested against *E. coli* (Gram-negative) and MRSA (Gram-positive) bacteria. The samples included pure PVA solution and PVA liquid bandages with different concentrations of CuI suspension. Firstly, both the *E. coli* and MRSA microorganisms were cultured in Mueller Hinton Broth (MHB) at 37 °C for 18 h. After, the bacterial solutions of *E. coli* and MRSA were adjusted to 0.5 McFarland standard solution using a densitometer, determined as 1.5 × 10^8^ CFU/mL. Then, the bacterial solutions were diluted with MHB to 5 × 10^5^ CFU/mL. Each sample contained 3 mL of 5 × 10^5^ CFU/mL diluted inoculum with continual turbulence at 37 °C for 0, 1, 3, 6, 12 and 24 h. At each time, 20 μL of each inoculum was brought out and replaced with normal saline solution. Eventually, 5 drops of 10 μL of each serial dilution were dropped on prepared Mueller–Hinton agar (MHA) in a Petri dish and incubated overnight in an incubator with controlled relative humidity and temperature of 37 °C. The number of growing bacterial colonies at each time point was counted, averaged and compared with the number of the control culture by estimating the CFU/mL values. The percentage of bacterial reduction was calculated following this equation:

$$\mathrm{Bacterial}\;\mathrm{reduction}\;(\%)=\frac{N_{\mathrm{control}\;}{-\;N}_{\mathrm{specimen}}}{N_{\mathrm{control}}}\;\times \;100$$

where N_control_ is the number of bacterial colonies in the control (CFU/mL); N_specimen_ is the number of bacterial colonies in the specimen (CFU/mL).

## Figures and Tables

**Table 1:** Zeta potential of synthesized CuI NPs.

Sample	Gallic Acid (GA) Contents (mM)	Zeta Potential (mV)	Average Value (mV)
CuI	0	−13.3	−13.47
		−13.6	
		−13.5	
2.5 GA-CuI	2.5	−18.8	−18.73
		−18.6	
		−18.8	
5.0 GA-CuI	5	−20.7	−20.47
		−19.8	
		−20.9	

**Table 2:** Minimum inhibitory concentration (MIC), minimum bactericidal concentration (MBC) and ratio of CuI NPs for each bacterial strain.

CuI Sample	Gallic Acid Concentration (mM)	MIC (mg/mL)		MBC (mg/mL)	
		*E. coli* (−)	MRSA (+)	*E. coli* (−)	MRSA (+)
CuI	0	8	16	32	32
2.5 GA-CuI	2.5	16	32	64	32
5.0 GA-CuI	5.0	16	32	64	32

**Table 3:** Wave numbers of FTIR results for PVA and PVA loaded with CuI suspension.

Assignment	Observed Wavelength (cm^−1^)			
	Pure PVA	PVA/CuI (0.095%)	PVA/CuI (0.190%)	Reference
OH stretching	3268.62	3328.42	3299.37	[34]
Asymmetric stretching of CH_2_	2940.72	2940.72	2941.86	[34]
Symmetric stretching of CH_2_	2911.56	2911.56	-	[34]
C=O carbonyl stretching	1653.96	1703.27	1698.17	[34]
CH_2_ scissoring	-	1447.77	1444.98	[34]
CH_2_ bending	1414.07	1419.38	1414.05	[34]
CH_2_ wagging	1328.07	1317.24	-	[34]
C-OH stretching in alcohol	1236.24	1239.7	1234.42	[34]
C-O stretching	1090.47	1088.07	1090.16	[34]
Stretching of C=O and bending of OH	1039.52	1042.26	1042.96	[34]
C-C stretching	842.91	796.64	797.43	[34]

## Data Availability

Not applicable.
